# Host gene–microbiome interactions: molecular mechanisms in inflammatory bowel disease

**DOI:** 10.1186/s13073-017-0459-4

**Published:** 2017-07-24

**Authors:** Hiutung Chu

**Affiliations:** California Institute of Technology, Pasadena, CA 91125 USA

## Abstract

Recent studies have identified links between host genetic variants and microbial recognition of the microbiome. Defects in host–microbiome interactions in individuals harboring inflammatory bowel disease risk alleles may result in imbalances of the microbial community, impaired pathogen clearance, and failure to sense beneficial commensal microbes. These findings highlight the importance of maintaining bi-directional communication at the mucosal interface during intestinal homeostasis.

## Gene–environment interactions in inflammatory bowel disease

Inflammatory bowel disease (IBD), encompassing Crohn’s disease (CD) and ulcerative colitis (UC), is an idiopathic, chronic intestinal inflammatory disease involving complex interactions between genetic and environmental factors. There is significant variability in symptoms and clinical manifestations, which may partly reflect the genetic heterogeneity of IBD susceptibility. Indeed, close to 200 IBD susceptibility genes have been identified thus far, though the lack of complete penetrance indicates that additional unknown factors are likely to be involved in pathogenesis [1]. Among the susceptibility genes are those involved in intestinal barrier function, innate immune recognition of microbial ligands, antigen presentation, T-cell immunity, autophagy, and other immune-mediated pathways [1].

The interplay between the gut epithelium and the microbiota has also been implicated as a contributing factor in disease pathogenesis, whereby aberrant interactions may result in inflammatory responses to commensal microbes [1]. It has become understood that the composition of the microbiota is important in directing both the local and systemic immune responses through which specific beneficial microbes can promote immune homeostasis and mucosal tolerance to dampen inflammation [2]. In IBD, reduced diversity of the microbiota has been observed, with enrichment of pro-inflammatory microbes and depletion of anti-inflammatory bacteria. While the gut microbiome is acquired from birth, and stabilizes in the first years of life [3], it also serves as an environmental sensor that interacts with the host to influence immune phenotypes. Moreover, the composition of the microbiota can be influenced by a variety of environmental factors, including diet [3], on both short and long timescales. Thus, the heterogeneity of the microbial composition and its shifts at taxonomic and functional levels, may serve as another variable in clinical manifestations, highlighting the complex interaction between host genetics and the microbiota. Recently, progress has been made in the identification of novel genetic variants associated with IBD and in understanding their relationship with the gut microbiome, revealing that the maintenance of bidirectional communication at the mucosal interface is critical for sustaining intestinal homeostasis.

## The role of host genetics in shaping the microbiota

The relationship between host genetics and the composition of the gut microbiota has been explored extensively [4, 5]. The heritability of the microbiome, and its subsequent contribution to host health, is governed both by extrinsic factors (for example, mode of delivery, breastfeeding, diet) and by host genetics. A recent study examined the fecal microbiota of over 400 twin pairs and revealed that monozygotic twins harbored more similar microbiomes than dizygotic twins, suggesting heritability of specific taxa within the microbiome [6]. In mouse models, knockouts of genes linked to IBD have demonstrated significant shifts in the microbial communities in the gut, termed dysbiosis, supporting the notion that host genetics are critical in shaping the gut microbiota [7, 8]. Nucleotide-binding oligomerization domain-containing protein 2 (*NOD2*), a cytosolic innate immune pattern recognition receptor and the first CD susceptibility gene identified, has been reported to play key roles in maintaining intestinal homeostasis through its detection of peptidoglycan from the microbiota. A large body of evidence has revealed a role for NOD2 in shaping the composition of the gut microbiota in IBD patients as well as in animal models [7], with a recent report showing that the gut microbiota is altered in *Nod2* ^*−*/*−*^ mice, specifically with an expansion of *Bacteroides vulgatus*, a pathobiont [8]. The gene encoding caspase recruitment domain 9 (*CARD9*), another CD susceptibility gene whose product interacts with NOD2, was recently found to impact the gut microbiota, whereby *Card9* ^*−*/*−*^ mice harbor a more pro-inflammatory population of microbes with impaired tryptophan metabolism [9]. Tryptophan metabolites generated by the microbiota have been shown to be important in the mucosal immune response, and activation of the aryl hydrocarbon receptor (AhR) by tryptophan metabolites can direct the production of IL-22, a cytokine important in intestinal homeostasis. Transfer of the microbiota from *Card9* ^*−*/*−*^ mice into wild-type germ-free mice resulted in increased susceptibility to colitis, delayed recovery from disease, and reduced IL-22 responses compared to wild-type controls [9]. Accordingly, supplementation with tryptophan-metabolizing *Lactobacillus* strains was sufficient to rescue susceptibility to colitis. These and other findings clearly show that innate immune sensors and response regulators, such as NOD2 and CARD9, respectively, limit the expansion of disease-promoting microorganisms in the gut, allowing beneficial microbes to thrive, and thus contribute to host health.

## Understanding how genetic variants are involved in microbial sensing in IBD

The close interaction between the host and the microbiota involves the shaping of the microbiota by the host and concurrent signaling from commensal microbes through immune-mediated recognition receptors to promote host tissue and immune development [2]. Maintaining this bidirectional communication is critical for supporting intestinal homeostasis. Yet, defects in host signaling pathways may result in increased susceptibility to microbial infections while at the same time may disrupt the fundamental interactions with the indigenous commensal microbes. Indeed, genome-wide association studies (GWAS) have identified susceptibility genes involved in immune signaling pathways [1]. As described above, NOD2 is involved in bacterial sensing and pathogen surveillance during health and disease. IBD patients harboring risk variants for *NOD2* are believed to be impaired in bacterial recognition and clearance, resulting in sustained intestinal inflammation—a notion supported by studies of *Nod2* ^*−*/*−*^ mice in response to bacterial infections [7]. Furthermore, NOD2 stimulation has been shown to activate autophagy, a conserved cellular pathway involved in the clearance of intracellular microbes [1, 7]. GWAS have also identified a link between autophagy and IBD, with autophagy pathway genes *ATG16L1* (autophagy-related 16 like 1) and *IRGM* (immunity-related GTPase M), along with *NOD2*, being associated with CD susceptibility [1]. Knockout mouse models for the genes *Atg16l1*, *Irgm*, or *Nod2* have all been reported to be impaired in pathogen clearance. Moreover, defects in ATG16L1 result in diminished secretion of antimicrobial peptides from abnormal Paneth cells and reduced antigen presentation—all features that may contribute to IBD pathogenesis [1].

In addition to the detection and clearance of intracellular pathogens, we recently revealed a role for ATG16L1 and NOD2 in sensing immunomodulatory commensal bacteria to promote tolerogenic immune responses to maintain intestinal homeostasis. Mouse dendritic cells deficient in either *Atg16l1* or *Nod2* were unable to induce regulatory T-cell responses to suppress mucosal inflammation upon treatment with *Bacteroides fragilis*, both in vitro and during experimental colitis [10]. Furthermore, immune cells from patients harboring the *ATG16L1* risk allele T300A were unable to respond to *B. fragilis* in vitro compared to immune cells carrying the protective T300 allele, indicating that a functional ATG16L1 protein is required for the induction of anti-inflammatory responses by immunomodulatory bacterial molecules. Therefore, in addition to their role in bacterial clearance, we have proposed a complementary role for ATG16L1 and NOD2, in which these immune pathways also participate in the sensing of beneficial microbes [10]. Our work suggests that patients carrying risk alleles of IBD susceptibility genes may be cumulatively affected, first in sensing and clearance of intracellular microbes, and second in the recognition of immunomodulatory bacterial molecules from the gut microbiota to promote immune tolerance. Collectively, dysbiosis of the microbiome and the loss of beneficial commensal microbes, along with impairments in these IBD susceptibility genes, may lead to chronic, sustained mucosal inflammation, contributing to IBD pathogenesis.

## Conclusions

While host genetics and the gut microbiome have separately been considered to be contributing factors to host health and disease in various immune-mediated pathologies, it is now clear that the synergism between these two players is key to our understanding of the pathogenesis of IBD. The intestinal microbial community has evolved alongside the host, contributing to immunity and energy metabolism [2]. Emerging studies have revealed that a more diverse microbiome and its microbial metabolites are important in maintaining intestinal homeostasis. However, dysbiosis may result in a loss of microbial functional pathways important in the production of bacterial metabolites, such as tryptophan [9], short chain fatty acids (SCFAs), and other compounds with anti-inflammatory activities to regulate intestinal inflammation [1]. Specifically, IBD patients have lower levels of tryptophan and SCFAs in serum and fecal samples [9]. Further work is necessary to determine if these bacterial metabolites may serve as biomarkers for IBD, and if supplementation of these missing bacterial populations (as probiotics) and/or metabolites may be sufficient to serve as a potential IBD therapy.

Exploring gene–gut microbiome interactions may uncover novel strategies for early diagnosis and treatment of IBD. As probiotics and fecal microbiota transplant therapies become increasingly prevalent for IBD treatment, there is also a need to understand the mechanisms by which host immune pathways integrate microbiome-derived signals to promote intestinal homeostasis. Understanding whether individuals carrying risk alleles associated with IBD are receptive to the beneficial effects of microbial therapies will facilitate personalized approaches to treatment. The implications of the microbiome on precision medicine may be an important consideration for treatment of IBD and other immune-mediated diseases. In fact, recent studies have shown that the gut microbiome can predict patient responsiveness to immunomodulatory therapies, including both chemotherapies and an anti-integrin therapy (vedolizumab) used in IBD [11]. A personalized medicine approach that takes into consideration both host genetics and the gut microbiome could serve to predict therapeutic responses in the treatment of IBD and other immune-mediated diseases. Furthermore, future studies to identify novel pathways and their interactions with microbial communities could provide targets for more effective therapies and a better understanding of the pathogenesis of IBD.

## Abbreviations

CD: Crohn’s disease
GWAS: Genome-wide association study
IBD: Inflammatory bowel disease
SCFA: Short-chain fatty acids
UC: Ulcerative colitis
